# Gunshot Wound Contamination with Squirrel Tissue: Wound Care Considerations

**DOI:** 10.1155/2014/342914

**Published:** 2014-01-22

**Authors:** Porter W. Maerz, Tricia B. Falgiani, Robyn M. Hoelle

**Affiliations:** Department of Emergency Medicine, University of Florida, 1329 SW 16th Street, P.O. Box 100186, Gainesville, FL 32610-0186, USA

## Abstract

While report of animal bites contaminating wounds is reported commonly, direct wound contamination with squirrel flesh has never been reported in the literature. The patient suffered an accidental self-inflicted gunshot wound that drove squirrel flesh and buck shot deep within his right buttock. This case outlines his hospital course and wound treatment. The patient was treated with ten days of broad spectrum antibiotics, extensive debridement of the wound in the operating room, and further treatment of the wound with a vacuum dressing system. While squirrel tissue and buckshot had to be removed from the wound on day six of the hospital stay, the patient remained afebrile without signs or symptoms of systemic illness.

## 1. Introduction

Penetrating injuries account for up to twenty percent of all pediatric trauma admissions, and gunshots are the most common mechanism of injury in these patients [1]. Traditionally, civilian gunshot wounds are not at high risk for infection, regardless of the projectile's passage through clothing. However, the inclusion of animal tissue inside of the wound itself is a unique consideration. Animal inflicted wounds, such as bites and abrasions, are a common occurrence, with an annual incidence of 1-2 million, and carry a risk for zoonotic infections [2]. In particular, squirrels, as part of the rodent family, carry the potential to harbor a high number of pathogens capable of infecting a human. While most infections from rodents occur via either direct contact (i.e., a bite) or due to indirect contamination (via an arthropod vector), gross contamination of an open wound with squirrel flesh is an unreported event [3].

## 2. Case Presentation

The patient was a teenage Caucasian male with no significant past medical history who arrived to the Emergency Department (ED) via ambulance with a complaint of gunshot wound to the right buttock approximately one hour prior to arrival. According to the patient he was using the butt of his 12 G shotgun to dislodge a dead squirrel from a branch over his head during a hunting trip and shot himself with a load of birdshot in the right buttock. He presented with stable vital signs and reported no pain other than at the wound.

**Figure 1:**
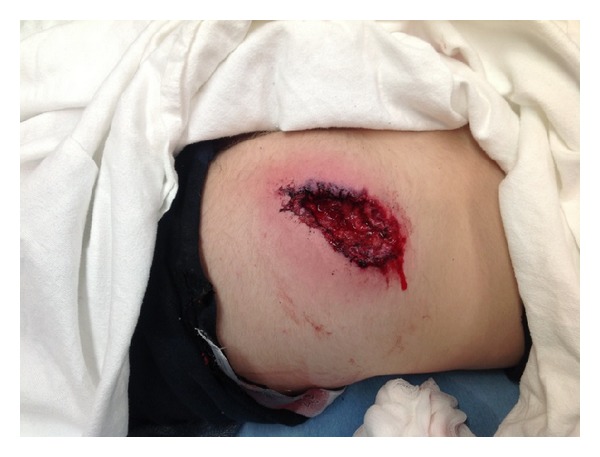
Wound on right posterior buttock.

On physical exam the patient appeared in no distress with mild tachycardia with a heart rate of 116. A 10 × 4 × 3 cm deep wound on the right buttocks was hemostatic (Figure 1). The edges of the wound were black and ragged, while there was circumferential surrounding erythema that extended 4 cm beyond the wound. Rectal exam revealed normal tone without gross blood and no palpable foreign bodies near the rectum. Debris was observed in the margin of the wound. The rural transporting EMS personnel promptly identified the material as “squirrel parts.”

**Figure 2:**
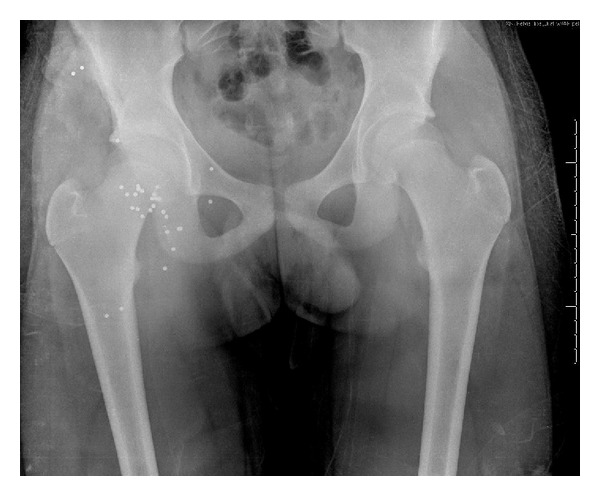
Anterior-posterior pelvic film demonstrating foreign bodies throughout the right buttock.

Copious wound irrigation with saline irrigation and debridement occurred in the emergency room, during which more pieces of animal flesh were found grossly contaminating the wound. There was also concern that the trajectory and final positioning of the buckshot in his buttock rested near the anus (Figure 2). Questioning of the patient revealed that the birdshot likely traveled through the rear pouch of his hunting vest which contained several squirrels killed earlier in the day.

After initial decontamination the patient was started on a ten-day course of IV piperacillin and tazobactam which is a fourth-generation, extended spectrum penicillin and a beta-lactamase inhibitor for prophylaxis. A pelvic X-ray revealed no signs of fracture but showed approximately 30 pellets of birdshot throughout the buttock. None of the metal appeared to have compromised the anus.

The patient was admitted to pediatric surgery service. The patient underwent six days of bed rest with a wound vacuum system (WVS) in place before being taken to the operating room (OR) for possible closure. The wound exhibited dusky edges further into the hospital stay and required further debridement and also required removing birdshot pellets and more pieces of squirrel tissue and fur. A WVS was replaced and three days later he underwent a delayed layered primary wound closure. His hospital course lasted 11 days during which he remained afebrile and stable. The patient was discharged without oral antibiotics and seen for followup two weeks later. At that time his wound was healing without complications or signs of infection and he was cleared for a return to normal activities. All cultures and pathology specimens were negative for bacterial growth.

## 3. Discussion

While penetrating trauma is less common than blunt trauma in the pediatric population, it still accounts for 10–20% of all pediatric trauma admissions [1]. Wounds caused by gunshots are the most common of this group. Shotgun injuries, while typically considered to be a low or medium velocity weapon, can cause massive tissue damage dependent on the range and weight of shot [4]. These injuries often require aggressive debridement, with removal of wound margins and careful inspection for foreign material carried by the shot, including clothing and shotgun wadding [5].

Antibiotic prophylaxis following gunshot wounds is still a debated topic. While bullets and their wounds are not sterile, several studies have shown that use of antibiotics does not affect the already low rate (1.8%) of infections in low-velocity injuries [6, 7]. In contrast to this, patients with high velocity injuries or fractures are at greater risk for infection due to the amount of soft tissue damage and potentially devascularized tissue [5]. The environment that civilian high velocity and shotgun injuries tend to occur in, rural or wooded areas, can increase the risk of environmental contaminants, including animal fecal matter [8]. In wounds that are grossly contaminated with dirt or clothing, the broad-spectrum antibiotic course has been recommended to extend for up to two weeks [5].

This case presents with the unique complication of having animal flesh carried into the wound by the birdshot. While the literature is silent on similar cases, the risk of transferring zoonotic pathogens cannot be ruled out. The literature is clear that zoonotic infections have been associated with squirrel bites. While rare, such bites have been associated with infections of rabies, tularemia, and typhus [9]. Rat bite fever has also been reported with squirrel bites, a systemic illness often caused by *Streptobacillus moniliformis* infection [10]. It must be assumed that contamination of an open penetrating wound with squirrel flesh has a risk of inducing similar zoonotic bacteria to the patient.

Rapid initiation of antibiotic prophylaxis has been shown to reduce the rate of infection due to animal bites in multiple studies and is particularly indicated in patients with deep penetrating wounds and those requiring surgical closure [8]. Use of an extended spectrum beta-lactam is often cited as appropriate prophylaxis for many types of bite wounds and is the standard choice in treating rat bite fever. This case report suggests that aggressive debridement and prophylactic antibiotics were successful in preventing infection from direct contamination of the wound by squirrel flesh.
